# 

**Published:** 1908-02-15

**Authors:** 


### ANNOUNCEMENTS.

THE NEW JERSEY STATE BOARD for Registration and Examinat on in Dentistry will hold its semi-annual meeting in the Assembly Chamber of the State House at Trenton, N. J., on Monday, July 6th, Tuesday, July 7th, and Wednesday, July 8th, 1908. All applications must be accompanied by a photograph and preliminary and professional requirements of the dental law, or the same wi l not be accepted.

For full particulars, apply to the Secretary,

CHARLES A. MEEKER, D. D. S.,
29 Fulton Street,
Newark, N. J.

SEMI-CENTENNIAL JUBILEE MEETING OF THE INDIANA STATE DENTAL ASSOCIATION.—The Indiana State Dental Association will celebrate its Fiftieth Anniversary June 4, 5 and 6, at Indianapolis, with one of the largest meetings ever held The State Associations of Michigan, Ohio, Kentucky and Illinois have accepted invitations to meet with us. There will be five essayists: Drs. G. V Black, Illinois; T. W. Brophy, Illinois; Charles Zederbaum, Michigan; M. H. Fletcher, Ohio and H. B. Holmes, Kentucky. There will be fifty clinicians from these four States and practically all other State Associations will be represented by clinicians.

All ethical dentists are invited, as this will be the big meeting of the year. Yours truly,

D. A. HOUSE.

MICHIGAN SECOND DISTRICT DENTAL SOCIETY.—Pursuant to a call issued by the Committee from the State Society on Reorganization, dentists of Washtenaw and Jackson counties, and from the towns of Albion, Homer and Marshall, in Calhoun county, met at Jackson January 14th, and agreed to organize a Society which shall meet four times a year to be known as the Second District Dental Society of Michigan. The Society met at Ann Arbor February 11th for election of officers and completion of organization. This ought to be a good, live dental society and it will doubtless stimulate all the dentists of this district to get into dental society work. There are about eighty dentists in the district. Three fourths of them ought to be expected to join the society, which will make a very respectable society.

ORTHODONTIST.—Dr. Joseph B. Stewart, of Dayton, Ohio, has sent out his cards announcing that hereafter he will confine his practice to Orthodontia.

FIFTY-SECOND ANNUAL MEETING MICHIGAN STATE DENTAL SOCIETY, June 10, 11, 12 and 13, 1908, aboard Steamer City of Mackinac, en route to the "Soo," Mackinac Island and return.

The much-talked of boat trip, in connection with the annual meeting of the Michigan Dental Society, is actually going to take place. The Committee having charge of arrangements have already secured the large passenger steamer City of Mackinac, which will sail on *Wednesday, June 10th*, 1908, at half-past nine in the morning. We will thus be able to spend the morning viewing the attractive scenery along the Detroit River, across Lake St. Clair, through the Flats, (the so-called Venice of America) and the St. Clair River, on to Port Huron, where the last stop to take on passengers will be made. Immediately after clearing this port the first meeting will be held, and after this a six o'clock dinner, followed by a smoker on deck and a short evening session. Table clinics will be the chief feature of the meeting, and ample time and opportunity for a leisurely and complete observation of all that is being done will be one of the advantages of the trip.

Early in the morning of the second day we will find ourselves entering St. Mary's River, and the entire morning will be devoted to the pleasure of viewing the scenes along this beautiful river, an inspection of the locks, and a couple of hours of sight-seeing at the Soo. After luncheon on board an afternoon session will be held, which we hope will be largely attended by those men in the northern part of the state who are unable to be with us on the entire trip. It is hoped to make this particular session the most important of the meeting, and one to be long remembered, and after dinner an evening session will be held while we are still in port.

The start on our return trip will be made early Friday morning, and everybody will be free to do as he pleases; an opportunity will also be given to spend a couple of hours viewing the sights of Mackinac Island. The final sessions of the Society will be held on Friday afternoon and Saturday morning. Friday evening being devoted to music, dancing, and a general good time. The boat will arrive in Detroit early enough Saturday afternoon to allow all members residing out in the State to get evening trains for home.

The expense of this trip, including passage, meals and berth, will be nineteen dollars for the round trip for each passenger. It will be necessary for those desiring to have accommodations reserved to send their names, together with a check for five dollars per person to Doctor Oliver W. White, 406 Fine Arts Building, Detroit, Michigan, on or before March 1st, 1908. Reservations will be made in the order in which requests are received, and your preference for either inside or an outside stateroom will be complied with so far as possible. The Navigation Company insists upon closing a contract with us by March 1st, 1908, and it is for this reason that we must have early replies, and a deposit of five dollars per passenger; for otherwise we would not be in position to assume the heavy financial obligation involved in chartering the boat. You are also requested to send a check for the balance of the amount on May 1st, and immediately upon the receipt of this the chairman will forward tickets for both passage and berth. Please state in your application for reservations, if more than one is asked for, whether it is for yourself and wife or for two men, and if for two men, who must be dentists, whether or not you are willing to have a third man assigned to the same stateroom. There is a double and a single berth in each stateroom, and because of the limited number of these rooms, (only 150) we will especially appreciate the courtesy if men unaccompanied by their wives are willing to go three in a room. We are compelled to limit family parties to three persons who are willing to occupy a single stateroom.

All ethical dentists are cordially invited.

Yours very truly,

Dr. Oliver W. White,
406 Fine Arts Bldg.,
Detroit, Mich.

